# Comparison of Conjunctival Flora Before and 12 Months After Dacryoendoscopic Recanalization for Lacrimal Passage Obstruction

**DOI:** 10.3390/jcm14217778

**Published:** 2025-11-02

**Authors:** Takahiro Hiraoka, Sujin Hoshi, Kuniharu Tasaki, Tetsuro Oshika

**Affiliations:** Department of Ophthalmology, Institute of Medicine, University of Tsukuba, Ibaraki 305-8575, Japan; hoshisujin@md.tsukuba.ac.jp (S.H.); tasaki.kuniharu.zh@ms.hosp.tsukuba.ac.jp (K.T.); oshika@eye.ac (T.O.)

**Keywords:** conjunctival flora, lacrimal drainage, microbiology, dacryoendoscopic recanalization, lacrimal passage obstruction, silicone tube, bacterial species, commensal bacteria, long-term, normalization

## Abstract

**Background/Objectives**: To investigate the long-term changes in conjunctival bacterial flora before and after dacryoendoscopic recanalization for lacrimal passage obstruction using silicone tube intubation. **Methods**: This prospective study included 135 eyes with lacrimal passage obstruction that underwent lacrimal passage recanalization and were followed for at least one year. The silicone tubes inserted during surgery were removed three months postoperatively in all cases. The study period was from November 2018 to January 2025. Conjunctival samples were obtained before surgery and at 12 months postoperatively. Aerobic cultures were performed to detect bacterial flora. The culture positivity rate, number of bacterial species identified, and proportion of commensal bacteria were compared before and after surgery. **Results**: The bacterial culture positivity rate significantly decreased from 36.3% preoperatively to 20.0% postoperatively (*p* = 0.003). The number of bacterial species detected decreased from 15 to 6, with Gram-negative bacilli decreasing from 6 species to 1. In contrast, the proportion of commensal bacteria such as coagulase-negative staphylococci and *Corynebacterium* spp. relatively increased from 49.1% to 80.7%. No drug-resistant bacteria were detected postoperatively. **Conclusions**: Dacryoendoscopic recanalization for lacrimal passage obstruction was shown to achieve long-term normalization of the conjunctival bacterial flora by reducing pathogenic and drug-resistant bacteria and increasing commensal bacteria. These findings suggest that the procedure prior to intraocular surgery in patients with lacrimal obstruction may reduce the risk of postoperative infection.

## 1. Introduction

Obstruction of the lacrimal drainage system is a common ophthalmic condition that can cause symptoms such as epiphora, recurrent conjunctivitis, and dacryocystitis. It is well established that such obstruction alters the conjunctival microbiota, often increasing the prevalence of pathogenic and drug-resistant organisms, thereby elevating the risk of postoperative endophthalmitis during intraocular procedures [1,2,3,4,5]. Therefore, preoperative management of nasolacrimal duct obstruction is crucial in patients scheduled for intraocular surgery.

Traditionally, external or endonasal dacryocystorhinostomy (DCR) has been the standard surgical treatment for nasolacrimal duct obstruction. While DCR effectively restores drainage, it does so by bypassing the physiological pathway of the lacrimal system. Several studies have reported that the conjunctival flora becomes similar to the nasal flora one year after DCR, differing from that of non-operated normal eyes [6,7]. In addition, Owji et al. demonstrated that the rate of bacterial isolation from the conjunctiva of operated eyes increased compared with that of the contralateral normal eyes one year after DCR [6].

In contrast, dacryoendoscopic recanalization with silicone tube intubation aims to anatomically and functionally reconstruct the natural tear drainage pathway. The development of dacryoendoscopes and techniques such as sheath-guided probing and intubation has enabled surgeons to directly visualize and treat obstructions throughout the lacrimal passage, from the punctum to Hasner’s valve, with minimal invasiveness [8,9,10].

In our previous prospective study, we assessed the short-term effects (four months postoperatively) of dacryoendoscopic recanalization on conjunctival bacterial flora [11]. That investigation revealed a significant reduction in the culture positivity rate, a marked decrease in the number of potentially pathogenic and drug-resistant bacterial strains, and a relative increase in indigenous, commensal organisms such as coagulase-negative staphylococci and *Corynebacterium* spp. These findings suggested that dacryoendoscopic recanalization contributes to the normalization of conjunctival flora in the short term.

While DCR has been studied for its long-term effects on conjunctival flora [3,4,5,6,7], there is a lack of data on how the conjunctival microbial environment evolves over a longer postoperative period following physiological lacrimal pathway reconstruction via dacryoendoscopy. In particular, the risk of retrograde contamination from the nasal cavity—an issue occasionally noted after DCR due to its direct anastomosis between the lacrimal sac and nasal mucosa—does not apply to dacryoendoscopic procedures, which preserve the one-way physiological flow of tears [6]. Thus, the long-term microbial outcomes following dacryoendoscopic recanalization may differ from those observed after DCR.

To our knowledge, no previous studies have evaluated the status of the conjunctival flora one year after dacryoendoscopic recanalization. Considering that microbial flora can undergo time-dependent changes after lacrimal surgery, a long-term assessment is essential to understand the durability and clinical relevance of microbial normalization achieved by this procedure [6,7,12]. The present study aims to investigate the long-term (12-month) changes in conjunctival bacterial flora in eyes with lacrimal passage obstruction after successful dacryoendoscopic recanalization with silicone tube intubation. By analyzing the culture positivity rate, species diversity, and prevalence of commensal versus pathogenic organisms one year after surgery, we seek to determine whether the microbial normalization observed in our previous 4-month follow-up study is sustained over time. These findings have direct implications for determining the optimal timing of intraocular surgery in patients with lacrimal drainage obstruction and for understanding the microbiological benefits of restoring physiological tear flow via endoscopic approaches.

## 2. Materials and Methods

This prospective study included consecutive adult patients with epiphora secondary to lacrimal drainage system obstruction, with or without mucopurulent reflux on lacrimal syringing, who underwent successful dacryoendoscopic lacrimal passage recanalization with silicone tube intubation between November 2018 and January 2025 at the Maruyama Eye Clinic. Surgical success was defined as both anatomical patency confirmed by dacryoendoscopic examination and functional resolution of epiphora after tube removal. Eligible patients were followed for at least 12 months postoperatively, and conjunctival culture testing was performed at both baseline and the 12-month visit.

The study protocol was approved by the University of Tsukuba Hospital Institutional Review Board and the study conduct adhered to the tenets of the Declaration of Helsinki. After the study protocol was fully explained, all the study subjects provided written informed consent to participate in the study.

### 2.1. Inclusion and Exclusion Criteria

We included patients with nasolacrimal duct obstruction and/or presaccal obstruction (punctal or canalicular). Exclusion criteria were: the presence of any extraocular diseases causing ocular infection, including acute dacryocystitis; lacrimal system tumor; previous trauma to the ocular or nasal regions; a history of congenital nasolacrimal duct obstruction or surgical treatments for lacrimal drainage disorders; the use of topical ocular medications; systemic immunosuppression; and active symptomatic infection elsewhere in the body. Patients were also excluded if lacrimal passage obstruction recurred within one year after surgery.

### 2.2. Surgical Procedures

Patients received no topical or systemic antibiotics for at least 2 weeks before surgery. All surgeries were performed by a single experienced surgeon (T.H.) using sheath-guided endoscopic probing (SEP) and sheath-guided intubation (SGI), as previously described [6,7,8,13]. In brief, after infratrochlear nerve block with 2% lidocaine, the nasal mucosa was anesthetized and vasoconstricted with gauze soaked in 4% lidocaine and 0.01% epinephrine placed in the inferior meatus. The canalicular system was irrigated with 4% lidocaine solution, and the puncta were dilated with a tapered dilator. A dacryoendoscope (LAC-06FY-H; Machida Endoscope Co., Chiba, Japan) equipped with a sheath (created from an 18-gauge plastic cannula; Terumo Co., Tokyo, Japan) was inserted into the common canaliculus to directly visualize the obstruction. Obstructed sites were widened under endoscopic view (SEP). Once the endoscope tip reached the nasal cavity and all blockages were eliminated, the sheath was left in place. A 105 mm Nunchaku-style silicone tube (LACRIFAST CL; ROHTO Pharmaceutical Co., Ltd., Osaka, Japan) was connected to the sheath and placed into the recanalized passage (SGI). The same procedure was then performed from the other punctum, and correct intranasal positioning of both tube ends was confirmed by a transnasal endoscope.

### 2.3. Postoperative Management

Postoperatively, patients instilled levofloxacin ophthalmic solution 1.5% (Cravit, Santen Pharmaceutical Co., Osaka, Japan) and fluorometholone ophthalmic suspension 0.1% (Flumetholon, Santen Pharmaceutical Co.) four times daily for 3 months. Follow-up visits were scheduled at 1 week, and at 1 and 2 months after surgery, with lacrimal syringing performed at each visit. In addition, the tube position was confirmed to be appropriate in all cases using nasal endoscopy at the one-month postoperative visit. The silicone tube was removed at 3 months, and topical medications were discontinued. Thereafter, patients were examined every 2–3 months until the 12-month visit, with patency of the lacrimal passage assessed at each follow-up.

### 2.4. Conjunctival Culture

Conjunctival samples were collected from the lower fornix of the operated eye using a sterile dry cotton swab without instilling topical anesthesia, avoiding contact with the lid margins or eyelashes [3,4]. Samples were obtained within 1 week before surgery and again at 12 months postoperatively. Each swab was immediately placed in 1 mL thioglycolate medium and sent to an external microbiology laboratory (Koto Microbiological Laboratory, Tokyo, Japan) for aerobic culture. Media included 5% sheep blood agar, chocolate II agar, bromothymol blue lactose agar, and OPAII agar. Plates were incubated at 33–37 °C for 48 h, after which colonies were identified and enumerated using standard bacteriological techniques.

### 2.5. Outcome Measures and Statistical Analysis

Primary outcome measures were the bacterial culture positivity rate, the number of bacterial species isolated, and the proportion of commensal organisms (coagulase-negative staphylococci and *Corynebacterium* spp.) among all isolates. Comparisons between pre- and postoperative data were made using the χ^2^ test, with *p* < 0.05 considered statistically significant. Statistical analyses were performed using SPSS, version29 (IBM Corp., Armonk, NY, USA).

## 3. Results

A total of 135 eyes from 135 patients (94 females and 41 males) were included in the analysis. The mean age at the time of surgery was 75.3 ± 10.3 years (range, 32–95 years). The distribution of obstruction sites identified by dacryoendoscopy included nasolacrimal duct obstruction in 97 eyes, canalicular obstruction in 90 eyes, and punctal obstruction in 17 eyes; some eyes had multiple obstruction sites (Table 1).

### 3.1. Bacterial Culture Positivity Rate

**Figure 1:**
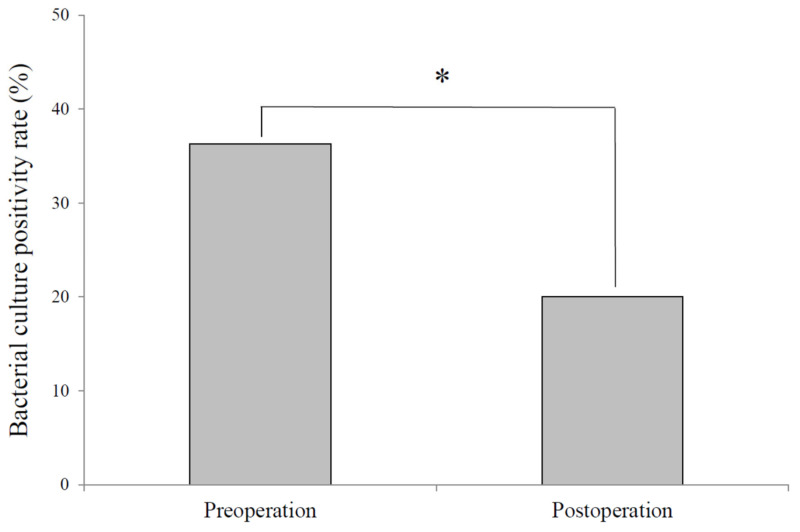
Change in bacterial culture positivity following surgery. Bacterial cultures yielded positive results in 49 of 135 eyes preoperatively, whereas only 27 of 135 eyes were culture-positive postoperatively, representing a significant reduction in bacterial isolation following surgery. *: Significant difference between pre- and post-operation by the χ^2^ test.

The overall bacterial culture positivity rate significantly decreased from 36.3% (49/135 eyes) before surgery to 20.0% (27/135 eyes) at 12 months postoperatively (χ^2^ test, *p* = 0.003) (Figure 1). This reduction was observed across all obstruction site categories.

### 3.2. Number and Types of Bacterial Species

**Figure 2:**
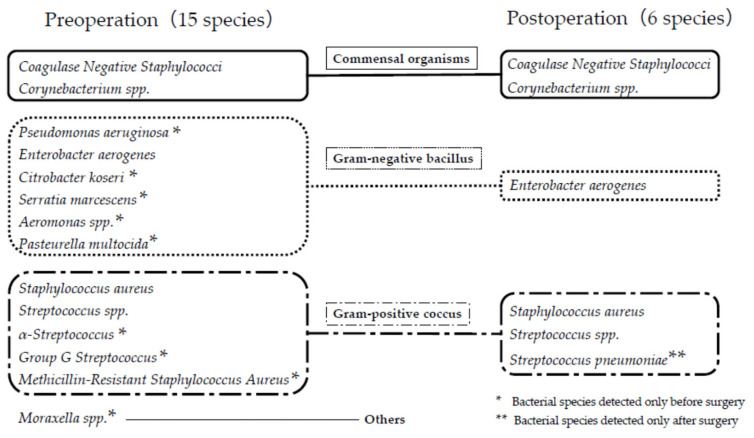
An Illustrative Figure of Overlap and Differences Between Pre and Post Surgery. The number of bacterial species isolated declined from 15 before surgery to six after surgery. Bacterial species detected only before surgery are indicated by a single asterisk (*), and those detected only after surgery are indicated by a double asterisk (**).

The total number of bacterial species isolated decreased from 15 species preoperatively to six species postoperatively. In particular, Gram-negative bacilli markedly decreased from six species before surgery to one species after surgery. Among Gram-negative bacilli, only a single strain of Enterobacter aerogenes was detected in one case, and the other five species were not detected at all (Table 2) (Figure 2).

### 3.3. Changes in Commensal Flora

**Figure 3:**
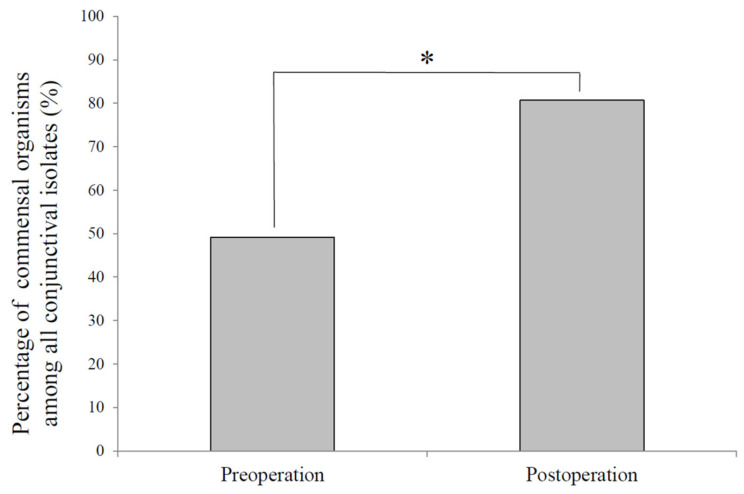
Percentage of commensal organisms among all conjunctival isolates before and after surgery. Coagulase-negative staphylococci and *Corynebacterium* spp., which are commensal organisms the in normal conjunctival flora, accounted for 49.1% of all isolates before surgery and 80.7% after surgery, representing a significant postoperative increase (*p* = 0.004, χ^2^ test). *: Significant difference between pre- and post-operation by the χ^2^ test.

The proportion of commensal organisms—coagulase-negative staphylococci (CNS) and *Corynebacterium* spp.—increased significantly from 49.1% of all isolates preoperatively to 80.7% postoperatively (*p* = 0.004) (Figure 3). This finding indicates a sustained normalization of the conjunctival bacterial flora one year after surgery.

### 3.4. Subanalysis Results During the COVID-19 Pandemic

An analysis of the 45 eyes treated during the COVID-19 pandemic period showed that the bacterial culture positivity rate decreased from 31.1% preoperatively to 25.0% postoperatively; however, this difference was not statistically significant (*p* = 0.227). The number of bacterial species detected decreased from five to three. The proportion of commensal bacteria, such as coagulase-negative staphylococci and *Corynebacterium* spp., increased from 50.0% to 90.0%, but this change was also not statistically significant (*p* = 0.350).

## 4. Discussion

This prospective study demonstrated that dacryoendoscopic recanalization with silicone tube intubation produces a sustained normalization of the conjunctival bacterial flora in eyes with lacrimal passage obstruction, maintained for at least 12 months postoperatively. The culture positivity rate, number of bacterial species, and prevalence of potentially pathogenic and drug-resistant organisms were all significantly reduced compared with the preoperative status, whereas the proportion of commensal bacteria markedly increased.

Our previous investigation, which assessed the short-term (4-month) microbiological effects of this procedure, showed similar trends, including a reduction in culture positivity rate from 42.0% to 26.0%, a decrease in the diversity of pathogenic microorganisms, and an increase in commensal flora [11]. The present study extends those findings, demonstrating that these beneficial alterations in conjunctival flora persist for at least one year after surgery.

Several previous studies have examined the effect of DCR on conjunctival flora [3,4,5,6,7]. While DCR can normalize conjunctival flora within weeks, some long-term follow-up studies reported partial reversion toward a nasal flora composition after one year, likely due to the direct anastomosis between the lacrimal sac and nasal cavity [6]. In contrast, dacryoendoscopic recanalization reconstructs the physiological lacrimal pathway from the punctum to Hasner’s valve, thereby preserving unidirectional tear flow and potentially avoiding retrograde contamination. The sustained postoperative microbial profile observed in our study supports this mechanistic advantage.

The marked decline in Gram-negative bacilli, including Pseudomonas aeruginosa, is also clinically significant. Gram-negative organisms are frequently associated with dacryocystitis and are more likely to cause aggressive postoperative infections [14,15]. Their substantial reduction after restoring physiological tear drainage underscores the role of tear flow in maintaining a healthy ocular surface microbiome.

As an additional analysis, we conducted a subanalysis of 45 eyes treated during the COVID-19 pandemic period. Similarly to the findings from the entire dataset, the bacterial culture positivity rate decreased, the number of bacterial species detected was reduced, and the proportion of commensal bacteria increased, showing a consistent trend; however, none of these changes reached statistical significance. This lack of significance is likely attributable to the reduced statistical power due to the smaller sample size, which was approximately one-third of the total cohort. The fact that only five bacterial species were detected preoperatively may suggest that the stay-at-home measures implemented during the COVID-19 pandemic could have influenced the composition of the bacterial flora, although this remains speculative. No other notable differences were observed.

This study has several limitations. First, we evaluated only aerobic bacteria; anaerobic cultures, including Cutibacterium species, were not performed and could provide additional insights. The exclusion of fungal and anaerobic organisms is particularly important in elderly populations, and this aspect was likely underrepresented in the present study. Second, cultures were obtained only at baseline and 12 months postoperatively, so the exact time course of microbial changes remains unknown. More frequent sampling could clarify how rapidly and stably flora normalization occurs. Third, we did not include a control group undergoing DCR, which would allow a direct comparison of long-term microbiological outcomes between these surgical approaches. Fourth, cases with recurrence within one year were excluded from the analysis. This may have introduced survivorship bias, potentially leading to an overestimation of the beneficial changes observed in the culture results. Fifth, the study was limited to a Japanese population; geographic and demographic differences in ocular flora may affect generalizability [16,17,18]. Finally, the postoperative use of topical levofloxacin and a steroid for three months may have exerted strong microbiological effects, which could have contributed, at least in part, to the favorable results observed in this study. Future investigations are needed to determine whether similar outcomes can be achieved with shorter postoperative use or without these medications.

## 5. Conclusions

Dacryoendoscopic recanalization with silicone tube intubation for lacrimal passage obstruction leads to significant and sustained normalization of the conjunctival bacterial flora for at least one year postoperatively. This includes a reduction in pathogenic and drug-resistant bacteria and an increase in commensal organisms. These findings have direct implications for the preoperative management of patients requiring intraocular surgery and support the use of physiological lacrimal pathway reconstruction to optimize ocular surface health

## Figures and Tables

**Table 1:** A summary of intraoperative diagnosis based on dacryoendoscopic findings.

Underlying Disease	Number of Cases
NDO only	47
NDO + CCO	41
CCO only	35
NDO + LCO	2
NDO + UPO	2
NDO + UPO + LPO	2
UPO + LPO	1
CCO + UPO + LPO	1
UCO + LCO + UPO + LPO	1
NDO + CCO + UCO + LCO	1
NDO + CCO + UPO + LPO	1
NDO + CCO + UCO + LCO + UPO + LPO	1
Total	135

NDO = Nasolacrimal duct obstruction, CCO = Common canalicular obstruction, UCO = Upper canalicular obstruction, LCO = Lower canalicular obstruction, UPO = Upper punctal obstruction, LPO = Lower punctal obstruction.

**Table 2:** A summary of conjunctival culture results.

	Microorganisms	Number of Isolates (Percentage of Total Isolates)				*p* Value
		**Preoperation**		**Postoperation**		
Gram-positive coccus	CNS	13	(21.7)	7	(22.6)	0.163
	*Staphylococcus aureus*	9	(15.0)	3	(9.7)	0.076
	*α-Streptococcus*	4	(6.7)	0	(0)	0.044 *
	*Streptococcus* spp.	2	(3.3)	1	(3.2)	0.562
	*Group G Streptococcus*	1	(1.7)	0	(0)	0.316
	MRSA	1	(1.7)	0	(0)	0.316
	*Streptococcus pneumoniae*	0	(0)	1	(3.2)	0.316
Gram-positive bacillus	*Corynebacterium* spp.	18	(30.0)	18	(58.1)	1.000
Gram-negative bacillus	*Serratia marcescens*	4	(6.7)	0	(0)	0.044 *
	*Pseudomonas aeruginosa*	2	(1.4)	0	(0)	0.156
	*Enterobacter aerogenes*	1	(1.7)	1	(3.2)	1.000
	*Pseudomonas putida*	1	(1.7)	0	(0)	0.316
	*Aeromonas* spp.	1	(1.7)	0	(0)	0.316
	*Pasteurella multocida*	1	(1.7)	0	(0)	0.316
	*Citrobacter koseri*	1	(1.7)	0	(0)	0.316
Others	*Moraxella* spp.	1	(1.7)	0	(0)	0.316
Total isolates		60	(100)	31	(100)	0.003 *

CNS = Coagulase-negative *Staphylococci*, MRSA = Methicillin-resistant *Staphylococcus aureus*; *: Significant difference between pre- and post-operation by the χ^2^ test.

## Data Availability

The data presented in this study are available on request from the corresponding author.

## Abbreviations

The following abbreviations are used in this manuscript:

DCR	dacryocystorhinostomy
SEP	sheath-guided endoscopic probing
SGI	sheath-guided intubation
MRSA	methicillin-resistant *Staphylococcus aureus*
CNS	Coagulase-negative Staphylococci
